# P-60. Lineage dynamics are not the major drivers of Streptococcus pneumoniae serotype 3 vaccine escape

**DOI:** 10.1093/ofid/ofae631.267

**Published:** 2025-01-29

**Authors:** Sydney Stanley, Catarina Silva-Costa, Joana Gomes-Silva, Richard Malley, Mario Ramirez

**Affiliations:** Boston Children's Hospital/Harvard Medical School, Boston, Massachusetts; Universidade de Lisboa, Lisbon, Lisboa, Portugal; Universidade de Lisboa, Lisbon, Lisboa, Portugal; Division of Infectious Diseases, Boston Children's Hospital, Boston, Massachusetts; Faculdade de Medicina, Universiudade de Lisboa, Lisboa, Lisboa, Portugal

## Abstract

**Background:**

*Streptococcus pneumoniae* serotype 3 (Spn3) is included in the pneumococcal 13-valent conjugate vaccine (PCV13) but carriage and invasive disease persist worldwide. Previous work showed that the prevalent clonal complex 180 (CC180) of Spn3 is comprised of 3 genetically distinct lineages: clade Iα, Iβ, and II; a post-PCV13 shift towards increased incidence of clade II strains with a concomitant decrease in clade Iα and 1β incidence has been observed in the United States, England, and Wales. It was hypothesized that PCV13 is controlling clade Iα and 1β but not clade II, resulting in clade II expansion and ultimately rendering the vaccine ineffective against Spn3. It is imperative to test this hypothesis in other regional contexts given the implications on vaccine design and efficacy. Therefore, our group assessed the dynamics of Spn3 population structure and PCV13 in Portugal.

Prevalence of Spn3 clonal complexes before and after the introduction of PCV13 in Portugal

**Figure:**
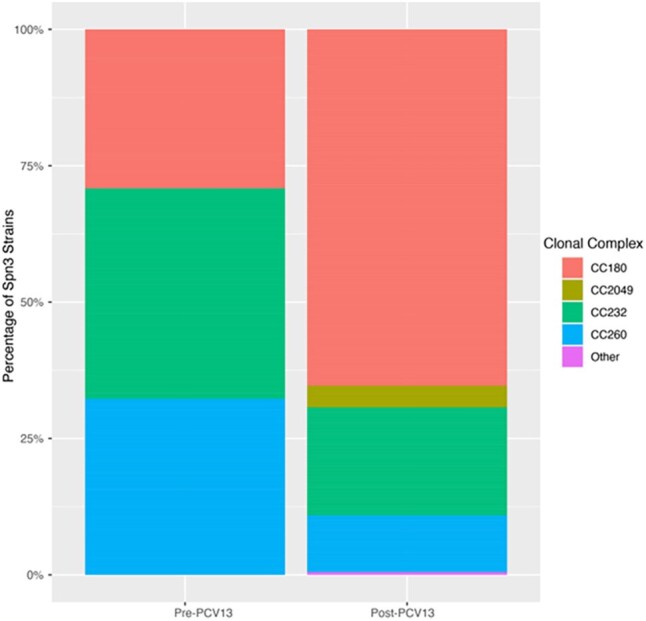

**Methods:**

We whole-genome sequenced 501 Spn3 strains from Portugal and analyzed over 1400 Spn3 whole genome sequences from published datasets for global comparisons. Phylogenetic tree construction, clade identification, clonal complex typing, and annotation of antimicrobial resistance (AMR) and virulence genes was used to evaluate changes in bacterial genetics.

Prevalence of Spn3 clades before and after the introduction of PCV13 in Portugal

**Figure:**
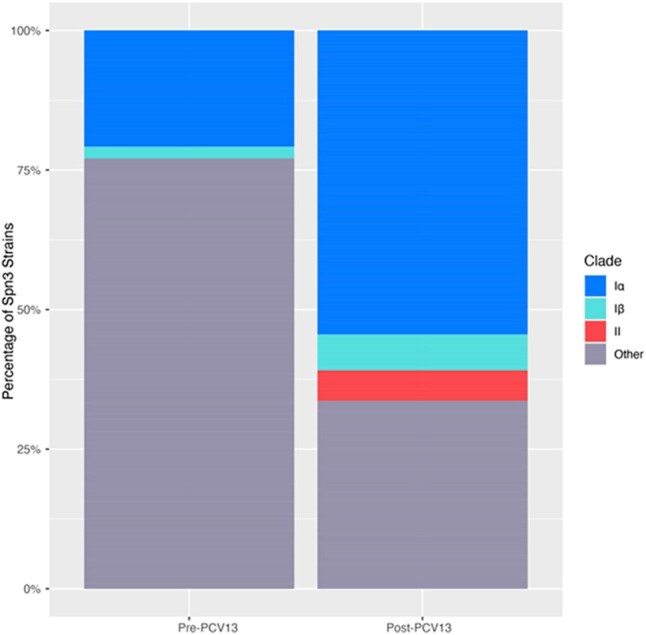

**Results:**

Consistent with previous work, the prevalence of clonal complexes significantly changed before and after the introduction of PCV13 in Portugal (p=4.94^-12^): CC180 increased from 29% of Spn3 strains to 65%. The prevalence of lineages also changed (p=2.33^-13^): clade Iα increased from 21% to 54% of Spn3 strains, while clade II was not detected pre-PCV13 but increased to only 5% post-PCV13. This contrasts with studies conducted elsewhere. The phylogenetic distribution of AMR alleles is similar across different regions, so altered antibiotic fitness does not explain the continued success of clade Iα over clade II in Portugal.

**Conclusion:**

Our data suggests clade II is not the global driver of Spn3 PCV13 vaccine escape as clade Iα remains dominant in Portugal. Current work aims to determine if clade Iα allelic variants augment virulence and thus competitive fitness against clade II in Portugal. Informed vaccine design requires an understanding of the region-specific bacterial population structure and genetic features to optimize efficacy.

**Disclosures:**

**Richard Malley, MD**, Amplitude Therapeutics: Board Member|Corner Therapeutics: Board Member|GSK: Advisor/Consultant|Merck: Advisor/Consultant **Mario Ramirez, PhD**, GSK: Advisor/Consultant|Merck Sharp and Dohme: Advisor/Consultant|Pfizer: Honoraria

